# A dynamic interaction model of topic interest and written language fluency: an empirical study

**DOI:** 10.3389/fpsyg.2025.1702699

**Published:** 2025-12-03

**Authors:** Jing Li, Fenghua Jin

**Affiliations:** School of Foreign Languages, Renmin University of China, Beijing, China

**Keywords:** topic interest, written language, lexical fluency, syntactic fluency, Chinese Spanish learners, complex dynamic systems theory

## Abstract

Drawing on dynamic systems theory (CDST), this study aims to explore how topic interest dynamically interacts with written language fluency among first-year Chinese university students majoring in Spanish. Using the moving correlation coefficient method, it investigates the evolving relationship between topic interest and lexical and syntactic fluency indicators across 18 essays per participant. A four-component analytical framework was applied, including (1) regression smoothing for trend identification, (2) correlation distribution analysis for significant associations, (3) fluctuation curve examination for temporal dynamics, and (4) change point detection for critical transitions. The findings reveal that the influence of topic interest on fluency is stage-dependent, with both positive and negative correlations coexisting. Notably, a high level of interest does not necessarily lead to fluent writing, and clear differences were found between lexical and syntactic fluency subsystems—lexical fluency tended to correlate positively with topic interest, while syntactic fluency showed negative correlations, reflecting uneven resource allocation between subsystems. This study extends CDST-based research in applied linguistics and psycholinguistics by revealing the nonlinear and multifaceted influence of affective factors on L2 writing. Pedagogically, the findings suggest that enhancing students’ topic engagement and balancing attention between lexical and syntactic fluency can foster more sustainable development in L2 writing.

## 1 Introduction

Writing is one of the most critical yet challenging skills to acquire in a second or foreign language (L2/ FL). It serves as a comprehensive indicator of a learner’s mastery of vocabulary, grammar, syntax, and cohesion. L2 writing is a complex cognitive process that involves planning, translation, execution, and monitoring (Kellogg, 1996). Successful writing relies on both general cognitive abilities, such as working memory, and language-specific cognitive abilities, including linguistic competence, to effectively process information and produce coherent texts at the levels of content, organization, and language (Kormos, 2012). Due to its cognitive and linguistic demands, L2 writing is widely regarded as the most difficult skill to master (Nosratinia and Razavi, 2016). To address this challenge, numerous studies have explored the dynamic development and variations in written language expression ability, as well as the underlying factors influencing these changes (Kowal, 2014; Wang et al., 2015; Rokoszewska, 2022; Li and Cai, 2022; Li, 2023, 2024a).

In today’s globalized world, effective communication through language has become increasingly essential (Biria and Jafari, 2013). This underscores the importance for learners to develop the ability to write and speak fluently in a second language (SL). Fluency, as a key feature of L2 development, reflects a learner’s capacity to generate and organize ideas while applying linguistic knowledge comprehensively (Qin and Bi, 2012). While extensive research has been conducted on oral fluency (Zhang, 2009; Foster, 2020; Suzuki and Kormos, 2023), recent years have seen a growing interest in written language fluency, with scholars exploring its dynamics and implications (Abdel Latif, 2013; Kowal, 2014; Pham, 2021; Tian et al., 2021).

Research on written language fluency has primarily focused on two key areas. On the one hand, studies have explored the predictive validity of various written language fluency parameters in relation to writing quality. For instance, Qin and Bi (2012) examined the predictive validity of frequency and ratio indicators, while Abdel Latif (2013) analyzed how written language fluency measurement has been influenced by oral production research, discussing the validity of time-related and finished product measures. On the other hand, a growing body of research has investigated the effects of different teaching interventions on written language fluency. For example, Alisaari and Heikkola (2016) explored the impact of singing as a pedagogical method on the development of second language learners’ written fluency. Similarly, Rezazadeh et al. (2011) studied the role of task type in shaping fluency, complexity, and accuracy in foreign language written production, and Pham (2021) focused on the effects of collaborative writing on students’ written language fluency, among other studies.

In recent years, the research paradigm of written language fluency has undergone significant transformation. This shift can be largely attributed to the introduction of complex dynamic systems theory (CDST) into L2 development research by Larsen-Freeman (1997) in the late 1990s. CDST challenged the traditional static research paradigms rooted in structuralism and generative grammar, offering a new lens to understand language development as a dynamic, nonlinear process. Furthermore, the application and rapid advancement of data visualization technologies in applied linguistics have facilitated interdisciplinary integration in L2 writing research. As a result, studies on written language fluency have increasingly embraced a dynamic, diachronic, and interaction-oriented approach, emphasizing variation and interconnectedness in language development (Chang and Chang, 2020).

And from the perspective of the psychology of language, L2 writing is not only a linguistic task but also a complex cognitive process involving the interaction of affective factors, working memory, and language production mechanisms. In the study of the dynamic development of written language expression ability, researchers (Wang et al., 2015; Li and Cai, 2022) have highlighted that students’ interest in topics—an affective variable tied to perceived value and emotional engagement—significantly influences the trajectory of changes in written language fluency indicators. However, there remains a notable gap in systematic research on the dynamic impact of topic interest on written language fluency, as well as the dynamic interaction patterns between the two. Furthermore, studies on the development of written language fluency among Chinese Spanish majors, particularly those grounded in CDST, are still scarce. Additionally, while the written language fluency system can be divided into various subsystems, no research has yet explored whether the influence of topic interest varies across these subsystems. In light of these gaps, this study was designed to systematically investigate the dynamic relationship between topic interest and written language fluency among L2 learners. Therefore, the present study addresses the following research questions:

1. What are the characteristics and main patterns of dynamic interaction between topic interest and written language fluency at the individual level?
2. How does the influence of topic interest vary across different subsystems within the written language fluency system?

## 2 Literature review

### 2.1 Complex dynamic systems theory (CDST)

Complex Dynamic Systems Theory (CDST) has its roots in Chaos Theory (Lorenz, 1963) and General Systems Theory (Bertalanffy, 1968). Larsen-Freeman (1997) introduced CDST into the field of applied linguistics to better understand and observe the dynamic processes underlying changes in learners’ language abilities over time. She emphasized that language systems are inherently dynamic, complex, and nonlinear, characterized by properties such as chaos, unpredictability, sensitivity to initial conditions, openness, self-organization, feedback sensitivity, and adaptability.

The characteristics of complex dynamic systems highlight that language systems consist of interconnected subsystems (van Geert, 1994), which continuously evolve and change over time, exhibiting varying degrees of variation at all levels. Their developmental trajectories are inherently complex, chaotic, and unpredictable (de Bot, 2008; Larsen-Freeman and Cameron, 2008). Moreover, the development of these systems and their subsystems is shaped by both internal learner factors and external environmental influences (Li, 2023, 2024a). These influencing factors are themselves interconnected and dynamically interact with the language system, forming an integral part of the L2 acquisition process (Larsen-Freeman and Cameron, 2008; Larsen-Freeman et al., 2011). Additionally, the subsystems within the language system engage in constant interaction and self-reorganization to adapt to changes in both internal and external conditions (de Bot et al., 2005).

Guided by CDST, research on L2 development emphasizes the interrelationships among components within the language system (Larsen-Freeman and y Cameron, 2013). It seeks to describe the emergent patterns that arise during individual development (Hiver et al., 2022) and the resulting complexity, dynamism, and nonlinearity that characterize the system’s evolution (Zhou, 2024). Within this framework, subsystems are intricately interconnected and mutually influential (de Bot et al., 2007; Larsen-Freeman and Cameron, 2008). These subsystems may exhibit competitive relationships, where one subsystem’s growth may coincide with another’s decline, or supportive relationships, where multiple subsystems develop simultaneously. The interplay of these subsystems drives changes within the broader language system (Feng et al., 2022). This interconnectedness is a core feature of complex dynamic systems, where changes in one subsystem can trigger significant variations in others or even the entire system—a phenomenon often referred to as the butterfly effect (Saadat and Alavi, 2017).

The complex system of L2 acquisition comprises multiple interconnected subsystems, including the learner, language, and environment (Li, 2024a). These subsystems interact in intricate ways to facilitate language learning (Larsen-Freeman, 1997; Dai and Wang, 2012). Within the learner subsystem, smaller components such as interest, motivation, attitude, personality, cognitive models, and learning strategies play critical roles (Larsen-Freeman, 1997). Recent studies have highlighted that high topic interest can significantly enhance students’ enthusiasm for written expression, thereby influencing the linguistic features of their written output (Wang et al., 2015; Li and Cai, 2022; Li, 2024a). As such, topic interest is a crucial socio-psychological factor that shapes the development of second language writing proficiency. Moreover, topic interest is an integral component of the L2 acquisition system. Investigating the dynamic interaction patterns between topic interest and the written language system—or its subsystems, such as fluency—can deepen our understanding of the self-organizing and adaptive characteristics of subsystems within the broader second language acquisition framework.

However, the current research model based on CDST still faces a significant limitation: the difficulty of balancing individual-level and group-level analyses. CDST places variability in language development at the core of its research agenda, as variability is often seen as an indicator of system evolution. The theory emphasizes the importance of individual differences and variation, considering them essential attributes of learners’ developmental trajectories (Zheng, 2019). Consequently, researchers have shifted their focus to individual-level variation, employing diachronic longitudinal designs to explore the development and interaction patterns within individual cases (Lowie, 2017). However, this approach often overlooks broader group-level trends and commonalities. Both individual variability and group commonality are critical to understanding language development processes (Spoelman and Verspoor, 2010). As Dörnyei (2014) pointed out, researchers must strive to balance the exploration of individual variability with the identification of group-level patterns. Similarly, Zheng and Li (2023) argue that an exclusive focus on individual analysis risks neglecting the emergence of overarching patterns. To address the current divide between individual differences and group-level generalizations, Lowie and Verspoor (2019) advocate for a mixed-methods approach that combines group statistics with case analysis. This approach uses group-level data to identify overall developmental trends and case studies to delve into individual variations, thereby providing a more comprehensive understanding of language development.

### 2.2 Written language fluency

Written language fluency can be defined in both narrow and broad terms (Qin and Bi, 2012). In the narrow sense, fluency refers to the ability to produce texts with ease and smoothness (Wolfe-Quintero et al., 1998; Hilton, 2008). This definition emphasizes time-related aspects, such as writing speed (Chenoweth and Hayes, 2001; Qin and Bi, 2012), vocabulary retrieval rate (Snellings et al., 2004; Qin and Bi, 2012), the number of words or language structures produced within a timed composition (Fellner and Apple, 2006; Qin and Bi, 2012), and the “automatic procedural skill that is relatively free from conscious attention” (Schmidt, 1992, p. 358). In the broader sense, written language fluency is often associated with a person’s overall language proficiency (Housen and Kuiken, 2009) and is considered a multidimensional construct. It is influenced by internal factors, such as the writer’s cognitive abilities, intuition, and imagination, as well as external factors, including context (Bruton, 1986). While there is no consensus in applied linguistics on a single definition of written language fluency, nearly all definitions emphasize core concepts such as efficiency, smoothness, effortlessness, and proficiency.

Based on differing conceptualizations of fluency, its measurement indicators can be categorized into two types: process indicators and product indicators (Abdel Latif, 2009). Process indicators focus on the writing process itself, employing methods such as video recording, think-aloud protocols, eye tracking, or keystroke logging to observe and analyze the entire writing process. These methods extract various metrics, including burst length (the number of words produced between pauses of 2 to 3 s), chunk length (the number of words written continuously without interruption), and processing load indicators (pauses longer than 200 milliseconds, pauses per minute, sentence fragments, etc.) (Qin and Bi, 2012; Van Waes, 2012; Ma and Qin, 2013). Research has demonstrated that process indicators effectively capture the characteristics of written language fluency and exhibit strong predictive power in distinguishing writing quality and language proficiency levels (Abdel Latif, 2009; Qin and Bi, 2012). However, despite their diagnostic value, process indicators are often limited in scope and complexity, making them challenging to apply in large-scale studies. Additionally, they may not provide a comprehensive understanding of the overall features of fluent texts (Qin and Bi, 2012).

Finished product indicators are derived from various metrics related to article quality, such as text length, accuracy, and complexity (Ma and Qin, 2013). These indicators can be further categorized into frequency indicators, ratio indicators, and overall scale indicators (Qin and Bi, 2012; Ma and Qin, 2013). Frequency indicators include measures such as total words, total verbs, total T-units, total clauses, total sentences, clauses per T-unit, total number of error-free T-unit words, and total number of error-free clause words (Ma and Qin, 2013). However, frequency indicators are highly sensitive to writing time and, as a result, tend to be less stable (Wolfe-Quintero et al., 1998). Ratio indicators, on the other hand, include metrics such as words per minute, average sentence length, average T-unit or clause length, length of compound noun phrases within T-units and clauses, and average length of error-free T-units and clauses (Ma and Qin, 2013). Unlike frequency indicators, ratio indicators are standardized measurements that are not influenced by text length. Consequently, they are better suited to reflecting developmental characteristics of language proficiency and distinguishing between different language levels (Qin and Bi, 2012).

The overall scale index refers to a composite measure designed to capture various aspects of language production, including authenticity, standardization, length, readability, and habitual usage (Tarone et al., 1993). However, this type of measurement is often criticized for its high degree of subjectivity and low reliability, which limits its practical application in research (Qin and Bi, 2012). As a result, overall scale indicators are rarely used in studies of written language fluency, with researchers typically favoring more objective and quantifiable metrics such as frequency and ratio indicators.

The parameters of written language fluency can be further categorized into lexical fluency indicators and syntactic fluency indicators. Lexical fluency, defined as the ability to produce vocabulary smoothly and accurately (Al-Abri et al., 2024), is often measured through indicators related to vocabulary retrieval efficiency (Pan et al., 2024). Within the framework of finished product fluency, metrics involving unit length—such as the average length of sentences, T-units, or clauses, as well as the average length of error-free T-units and clauses—fall under the domain of lexical fluency. Empirical studies have demonstrated that these unit length indicators are strongly correlated with writing quality (Wolfe-Quintero et al., 1998). For instance, Johnson et al. (2012) identified mean sentence length as a widely used and highly efficient indicator in empirical research on L2 written language fluency. Similarly, Qin and Bi (2012) found that the length of sentences, clauses, T-units, error-free clauses, and error-free T-units effectively distinguishes students across different proficiency levels and serves as reliable predictors of writing quality.

Syntactic maturity indicators are widely used to reflect syntactic fluency (Mellon, 1967). Syntactic fluency can be defined as the ability to produce syntactic structures, including complex constructions, with ease and flexibility (Shapiro, 2011). According to Smit (2004), metrics such as T-units, clauses, and related subordination indicators are effective in characterizing syntactic fluency. Consequently, frequency indicators such as total T-units, total clauses, total sentences, and clauses per T-unit within the finished product index are classified as syntactic fluency indicators. McAndrew (1990) employed these frequency indicators—total clauses, total T-units, and clauses per T-unit—as parameters for syntactic fluency and validated their effectiveness in measuring syntactic development.

### 2.3 Research on written language fluency based on CDST

Since the introduction of CDST into the field of applied linguistics, several studies have explored the dynamic development of fluency within the complexity, accuracy, and fluency (CAF) framework. For instance, Larsen-Freeman (2006) investigated the development of written language among five Chinese learners of English over a six-month period, adopting a CDST perspective. Participants were required to write a narrative on the same topic every month and a half. Written language fluency was measured using the average number of words per T-unit. The findings revealed a significant improvement in the group’s average written language fluency over time. However, notable individual differences were observed, with each learner exhibiting distinct developmental trajectories that diverged from the group trend.

Similarly, Saadat and Alavi (2017) conducted a study tracking and comparing the development of CAF parameters in individual writing versus collaborative writing among six Iranian college students over the course of a semester. Fluency was assessed using metrics such as the average number of words, T-units, and clauses per text. The results indicated that individual writing led to more fluent written language compared to collaborative writing.

In another longitudinal study, Kowal (2016) examined changes in the CAF of Swedish written language among 15 Polish college students over a three-year period. The study focused on fluency dimensions such as automaticity, rapidity in text production, and smoothness. The development of fluency parameters was characterized by chaos, unpredictability, and sensitivity to initial conditions. The three fluency subsystems—automaticity, rapidity, and smoothness—were found to be interconnected and mutually influential, yet they exhibited distinct developmental patterns. These patterns alternated between attraction states (relatively stable phases) and repulsion states (phases of intense fluctuation).

Other studies have focused exclusively on the development of written fluency. For example, Baba and Nitta (2014) investigated the development of written fluency in two English learners at a Japanese university over the course of an academic year. Written fluency was measured by counting the number of words produced in timed compositions. The findings revealed that fluency development followed a nonlinear trajectory. Both learners experienced at least one phase transition, but the timing of these transitions varied between individuals.

Likewise, Kowal (2014) conducted a longitudinal study tracking the fluency development of Swedish learners in Poland over a three-year period. The study employed two fluency parameters: mean transition time and mean length of burst. The results demonstrated that fluency development was nonlinear, unpredictable, and highly variable. Interestingly, the study found that writers with slower typing speeds did not necessarily exhibit lower written language fluency. Additionally, learners who showed slower initial progress often achieved higher levels of fluency by the end of the tracking period. Furthermore, students who began with slower typing speeds and lower fluency levels demonstrated the most significant improvements in fluency over the three-year period.

In addition, some scholars have explored the dynamic development of written fluency in the context of Spanish as a L2. For instance, Li and Cai (2022) investigated the development and changes in written CAF among Chinese Spanish majors during their first year of study. The findings revealed that fluency development generally followed an upward trend, characterized by a dynamic and nonlinear trajectory with alternating peaks and troughs. Specifically, the first semester marked a period of rapid improvement in fluency, while the second semester saw a transition into a relatively stable state. Key factors contributing to inter-individual variation included performance in small tests, students’ interests, logical reasoning abilities, the transfer effect of Chinese, and mastery of grammar.

Building on this, Li (2024a) examined the impact of learning environment changes on the development of written fluency among first-year Spanish majors in China. The study compared group and individual fluency trends when the learning environment shifted from classroom instruction during the semester to self-directed study during holidays. The results indicated that group fluency continued to exhibit a fluctuating development trend, with the overall trajectory remaining largely unaffected by changes in the learning environment. At the individual level, however, shifts in the learning environment led to varying degrees of quantitative changes in students’ learning beliefs, writing motivations, and attitudes, which in turn influenced the development trends of fluency. Both studies by Li and Cai (2022) and Li (2024a) operationalized written language fluency using the average length of T-units as the primary metric.

The studies reviewed above demonstrate that a considerable body of research has explored the dynamic development and changes of written fluency within the framework of CDST. However, several limitations remain. First, the fluency parameters examined in these studies are relatively simplistic, with most focusing on a single dimension, such as T-unit length or sentence length (e.g., Larsen-Freeman, 2006; Baba and Nitta, 2014; Li and Cai, 2022; Li, 2024a). Second, there is a scarcity of research focusing on Chinese Spanish majors, despite the fact that approximately 6,000 university students in China choose to study Spanish language and literature each year. Investigating the development and changes in their written fluency could offer valuable insights for Spanish language education and hold significant practical relevance. Finally, when analyzing the factors influencing written fluency, many studies adopt a subjective perspective, lacking quantitative and objective assessments (e.g., Wang et al., 2015; Li and Cai, 2022; Li, 2024a). Additionally, the scope of these influencing factors is often too broad, and the analysis tends to lack depth.

To address these gaps, it is essential to further deepen the application of CDST in studying the written language fluency of Chinese Spanish majors. This includes expanding the dimensions of fluency research, examining the dynamic impact of specific influencing factors on different aspects of fluency, and enriching research methodologies to achieve an effective integration of quantitative and qualitative approaches.

## 3 Methodology framework

### 3.1 Participants and study corpus

This study tracked the development of Spanish written language fluency among 18 Spanish majors at a public university in China during their first year of college. The participants, who enrolled in 2022, were all aged 17 or 18, comprising 16 female and 2 male students. All participants were native Mandarin Chinese speakers with no prior knowledge of Spanish. Although the sample size is relatively small (*n* = 18), it is sufficient for capturing individual-level variability within the CDST framework.

During the first semester, the students took the following courses: Basic Spanish I (8 h per week), Spanish Grammar I (2 h per week), Spanish Speaking I (2 h per week), and Spanish Phonetics (2 h per week). In the second semester, their coursework included Basic Spanish II (6 h per week), Spanish Grammar II (2 h per week), Spanish Speaking II (2 h per week), Spanish Listening I (2 h per week), and Spanish Reading I (2 h per week). The total teaching time remained consistent across both semesters at 14 h per week. The author of this study served as the instructor for Basic Spanish I and II.

Starting from the fifth week of the first semester, once the students had acquired basic writing skills, the author assigned them a timed narrative essay as weekly homework. Each timed narrative essay required participants to write at least 100 words within 40 min. Each week, the essay topic varied, with examples including “An Unforgettable Trip,” “A Day at Home,” “A Story Between Me and My Friends,” “A Happy Experience,” and “A Novel Shopping Experience.” Over the course of the first semester, each student completed 7 essays, and in the second semester, they completed 11 essays, resulting in a total of 18 essays per student by the end of the academic year. At the end of the first academic year, the author obtained consent from all 18 students to use their essays as the research corpus.

### 3.2 Study variables

This study focuses on the textual characteristics of fluency, investigating the dynamic interaction between fluency and topic interest at both lexical and syntactic levels. Given the extensive empirical support for T-units as a fundamental unit for measuring language proficiency (e.g., Wang et al., 2015; Li and Cai, 2022; Li, 2023, 2024a), this paper employs T-unit-based fluency indicators. Specifically, lexical fluency is measured using T-unit length (TL) and error-free T-unit length (EFTL), while syntactic fluency is assessed through Total T-units (TT) and clauses per T-unit (CPT). Drawing on established research (e.g., Larsen-Freeman, 2006; Johnson et al., 2012; Qin and Bi, 2012), the calculation formulas for these lexical and syntactic indicators are as follows:

Regarding topic interest, this study adopts the questionnaire developed by Schiefele and Krapp (1996) on topic interest in reading activities. According to these scholars, topic interest is divided into two dimensions: feeling-related valences and value-related valences. The first dimension captures students’ emotional responses to the topic, such as feeling “bored,” “stimulated,” “interested,” “indifferent,” “involved,” or “engaged,” as well as the intensity of these feelings. The second dimension reflects the perceived importance of the topic to the reader, which can be described using adjectives such as “meaningful,” “unimportant,” “useful,” or “worthless” (Schiefele and Krapp, 1996).

To evaluate the degree of emotion and importance, the study employs a four-point Likert scale adapted from Sadeghpour (2013), with response options ranging from “not true at all” (1 point) to “completely true” (4 points). However, the items “bored” and “indifferent” in the feeling dimension, as well as “unimportant” and “worthless” in the value dimension, are reverse-coded. For these four items, “not true at all” corresponds to 4 points, while “completely true” corresponds to 1 point.

In this study, students rated the 18 writing topics based on the ten interest parameters described above. The total interest score for each topic was calculated as the sum of all parameter scores.

### 3.3 Data analysis

This study employs moving correlation, a classic data analysis method under the CDST framework, to examine the dynamic relationship between topic interest and fluency indicators at both lexical and syntactic levels. To illustrate, the analysis of the correlation between topic interest and the lexical fluency indicator T-unit length (TL) is described as follows: a moving window is established for every five sets of data, resulting in a total of 14 moving windows across the 18 data points. The first window includes data points 1 to 5, the second window includes points 2 to 6, the third window includes points 3 to 7, and so on, until the fourteenth window, which comprises data points 14 to 18. Within each moving window, the correlation coefficient between topic interest and T-unit length is calculated, ultimately forming a moving correlation coefficient development curve.

The software Palabra XYZ is used to automatically generate the moving windows and compute the moving correlation coefficients between the two parameters. Previous studies (e.g., Li and Cai, 2022; Li, 2023, 2024a) have demonstrated the effectiveness of Palabra XYZ in processing such data. All analyses were cross-validated manually to ensure the reliability of automatically generated correlation values.

It is important to note that, due to the lack of variation in topic interest across multiple consecutive writing activities for Student D, the correlation coefficients for several moving windows could not be calculated. As a result, Student D’s data were excluded from the analysis, and only the trends of the remaining 17 students were examined.

In addition, to visualize the overall trend of the correlation, we employ regression smooth curves to observe the primary patterns of change. Furthermore, we utilize the Change-Point Analyzer to determine whether the correlation develops in distinct stages and to identify the specific locations of change points. Both the regression smooth curve and the Change-Point Analyzer are well-established and effective data analysis methods within the CDST framework, as demonstrated in previous studies (e.g., Baba and Nitta, 2014; Li, 2024a).

## 4 Results and discussions

The interaction patterns between topic interest and fluency parameters exhibit several key characteristics: (1) the development curve demonstrates continuous and complex changes; (2) the influence of topic interest reaches a statistically significant level; (3) the fluency system exhibits both synergistic effects and internal differences; and (4) the development process is characterized by distinct stages.

### 4.1 Continuous and complex changes

**Figure1:**
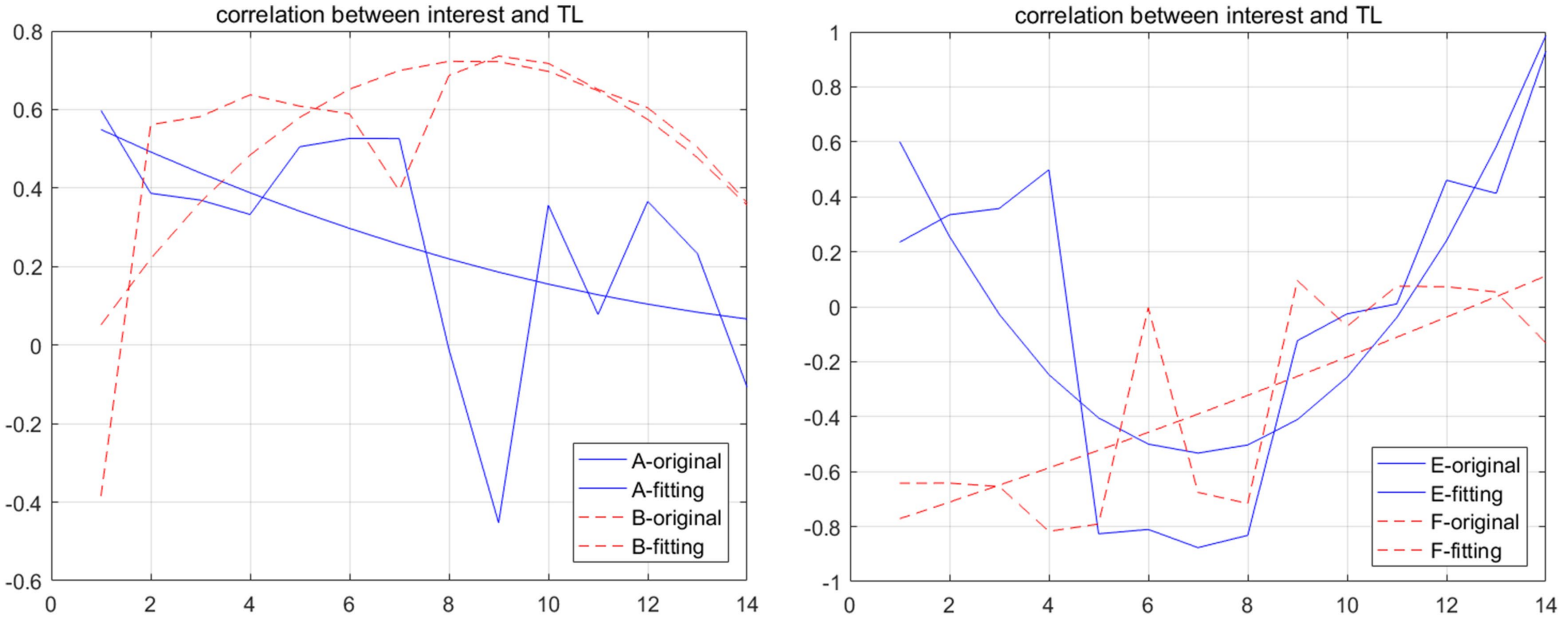
Development curve of the correlation between interest and TL of students A, B, E, and F.

Figure 1 illustrates the development trajectories of the correlation between topic interest and TL for four students: A, B, E, and F. The smooth curve represents the overall trend of each student’s correlation over time, while the fluctuation curve depicts the specific correlation values within each moving window. These four students were selected for display primarily because their overall trends exhibit distinct patterns, highlighting the variability in how topic interest and lexical fluency interact across individuals.

As illustrated in Figure 1, the overall trends of the moving correlation coefficients between topic interest and the fluency indicator TL can be categorized into four distinct patterns: (1) decline, (2) rise, (3) decline followed by a rise, and (4) rise followed by a decline. For instance, Student A exhibited a declining trend, characterized by a gradual weakening of the positive correlation. Student B demonstrated an initial rise followed by a decline, with the positive correlation reaching its peak before decreasing. Student E displayed a trend of decline followed by an increase, where the positive correlation transitioned into a negative correlation and then reverted to a positive one. Finally, Student F showed an overall upward trend, marked by a weakening of the negative correlation over time (see Table 1).

**Table 1:** Fluency indicators.

Indicator types	Indicators	Calculation formulas
Lexical fluency	T-unit length (TL)	total number of words/total number of T-units
	Error-free T-unit length (EFTL)	total number of words in error-free T-units/total number of error-free T-units
Syntactic fluency	Total T-units (TT)	Total number of T-units in the timed composition
	Clauses per T-unit (CPT)	Total number of clauses/total number of T-units

Four general trends were also observed in the correlation between topic interest and the other three fluency parameters, as shown in Table 2. The distribution of students across these trends varies significantly among the four correlations. Specifically, the relationship between topic interest and TL is primarily characterized by a downward trend and a downward trend followed by an upward trend. In contrast, the relationships between topic interest and EFTL, as well as between topic interest and TT, are mainly marked by an upward trend followed by a downward trend and a downward trend followed by an upward trend. Meanwhile, the relationship between topic interest and CPT is predominantly defined by a downward trend and an upward trend followed by a downward trend. These differences in the development patterns across the fluency subsystems reflect the CDST assertion that subsystems develop unevenly and exhibit distinct trajectories (Zheng and Feng, 2017; Li, 2023).

**Table 2:** Summary of overall change trend distribution.

Variable correlation pair	↓	↑	↓↑	↑↓
interest & TL	6	3	6	2
interest & EFTL	4	2	6	5
interest & TT	1	3	6	7
interest & CPT	6	3	2	6

The fluctuation curve in Figure 1 reveals that the correlation between topic interest and the internal components of the fluency system undergoes continuous and complex changes, with positive and negative correlations alternating over time. A positive correlation suggests that an increase in topic interest promotes the development of fluency components, whereas a negative correlation indicates that an increase in topic interest may inhibit the growth of the fluency system. For example, in the case of Student E, the correlation between topic interest and TL shows a positive correlation in moving windows 1–4, shifts to a negative correlation in windows 5–10, and then returns to a positive correlation in windows 11–14. This pattern aligns with previous research (Zheng and Feng, 2017), which highlights the alternating supportive and competitive relationships between subsystems within the language system. It also reflects the staged development of topic interest’s influence on the fluency system.

Furthermore, these findings underscore that topic interest is not the sole influencing factor; its impact on the fluency system does not exhibit a straightforward causal relationship but instead demonstrates the nonlinear development characteristics typical of complex dynamic systems (Verspoor et al., 2008; Waninge et al., 2014; Li, 2024b). This is because both the fluency system and the language acquisition system are complex dynamic systems influenced by numerous factors. For instance, students’ learning motivation, interests, psychological and physiological states during writing, and learning conditions can all significantly affect writing performance (Li and Cai, 2022; Li, 2024a). Consequently, even when students exhibit high topic interest, it does not necessarily translate into more fluent essays.

From a psycholinguistic view, the ongoing fluctuations and alternations in the correlation between topic interest and fluency components reflect dynamic adjustments of cognitive resource allocation in L2 writing. Per Kormos’ (2012) resource allocation model, learners’ limited working memory requires balancing resources across tasks like lexical retrieval (automatic, familiarity-driven: Schmidt, 1992) and syntactic encoding (controlled, rule-integrated). High topic interest may prioritize lexical retrieval, squeezing resources for syntactic encoding and reversing correlations; moderate interest may shift resources back to syntax. These fluctuations are not random but a manifestation of learners’ dynamic balance between automatic and controlled processes, reinforcing L2 writing as a multi-task cognitive coordination process.

### 4.2 Significant correlation between topic interest and fluency parameters

Regarding the correlation between topic interest and the fluency system, most students demonstrated a significant level of association. Table 3 provides a detailed breakdown, including: (1) the total number of students who achieved a significant correlation across the four fluency dimensions; (2) the number of students who exhibited only a significant positive correlation; (3) the number of students who exhibited only a significant negative correlation; and (4) the number of students who displayed both significant positive and negative correlations at different stages.

**Table 3:** Summary of distribution of significant correlations.

Fluency-interest dimension	Variable correlation pair	Significant correlation	Positive	Negative	Positive and negative
Interest & lexical fluency	Interest & TL	13	9	2	2
	Interest & EFTL	10	6	4	0
Interest & syntactic fluency	Interest & TT	13	2	9	2
	Interest & CPT	12	5	7	0

Among the students whose correlation between topic interest and lexical fluency reached a significant level, most exhibited a significant positive correlation, a smaller number showed a negative correlation, and very few displayed both positive and negative correlations. In contrast, among the students whose correlation between topic interest and syntactic fluency reached a significant level, most demonstrated a significant negative correlation, followed by a positive correlation, and finally a combination of both.

These results reveal two key findings. First, the lexical and syntactic fluency subsystems exhibit different tendencies in their significant correlations with topic interest. Specifically, more students showed a positive correlation in the lexical subsystem, while more students showed a negative correlation in the syntactic subsystem. This suggests that, in their first year of study, most students prioritize leveraging topic interest to enhance lexical fluency development rather than syntactic fluency.

This aligns with the assertion that the dynamic interaction patterns between various factors in second language acquisition systems are distinct (e.g., Li, 2023; Zheng and Feng, 2017) and supports the claim that language system resources are unevenly distributed in the early stages of learning, with development initially focused on a specific subsystem (e.g., Li and Cai, 2022; Li, 2023, 2024a). Therefore, it is crucial to strengthen syntactic fluency input during the first year of study. Educators should encourage students to focus not only on improving lexical fluency but also on developing syntactic fluency, particularly by producing longer sentences and utilizing clauses effectively.

Second, over the one-year tracking period, most students exhibited only one significant correlation (either positive or negative), while very few displayed alternating significant positive and negative correlations. For students who demonstrated both types of significant correlations, the magnitude of change in correlation coefficients was larger, and the cycles of change were shorter. This suggests that to determine whether alternating significant positive and negative correlations are a common phenomenon, it is necessary to extend the tracking period and observe the patterns in a broader sample of students.

Additionally, although most students showed significant correlations between topic interest and fluency, the number of moving windows with significant correlations in specific dimensions was relatively limited. For instance, in the four dimensions of correlation between topic interest and TL, EFTL, TT, and CPT, the average number of moving windows with significant correlations was 2.46, 2.2, 2.31, and 2.08, respectively. This indicates that the influence of topic interest on the fluency system is not consistently prominent but rather manifests in specific periods where it either promotes or inhibits fluency development. These findings further underscore the complexity of fluency as a dynamic system and the nonlinear nature of its interaction with influencing factors.

The stronger positive correlation between topic interest and lexical fluency, paired with a negative correlation with syntactic fluency, aligns with psycholinguistic models of resource allocation during L2 writing (Kormos, 2012). For first-year Spanish majors, high topic interest may direct limited working memory resources toward lexical retrieval—an automatic, familiarity-driven process—at the expense of syntactic encoding, which requires more effortful integration of grammatical rules. This pattern highlights how affective factors can modulate the trade-off between automatic and controlled cognitive processes in L2 production.

### 4.3 The synergy and diversity of the influence of topic interest

**Figure 2:**
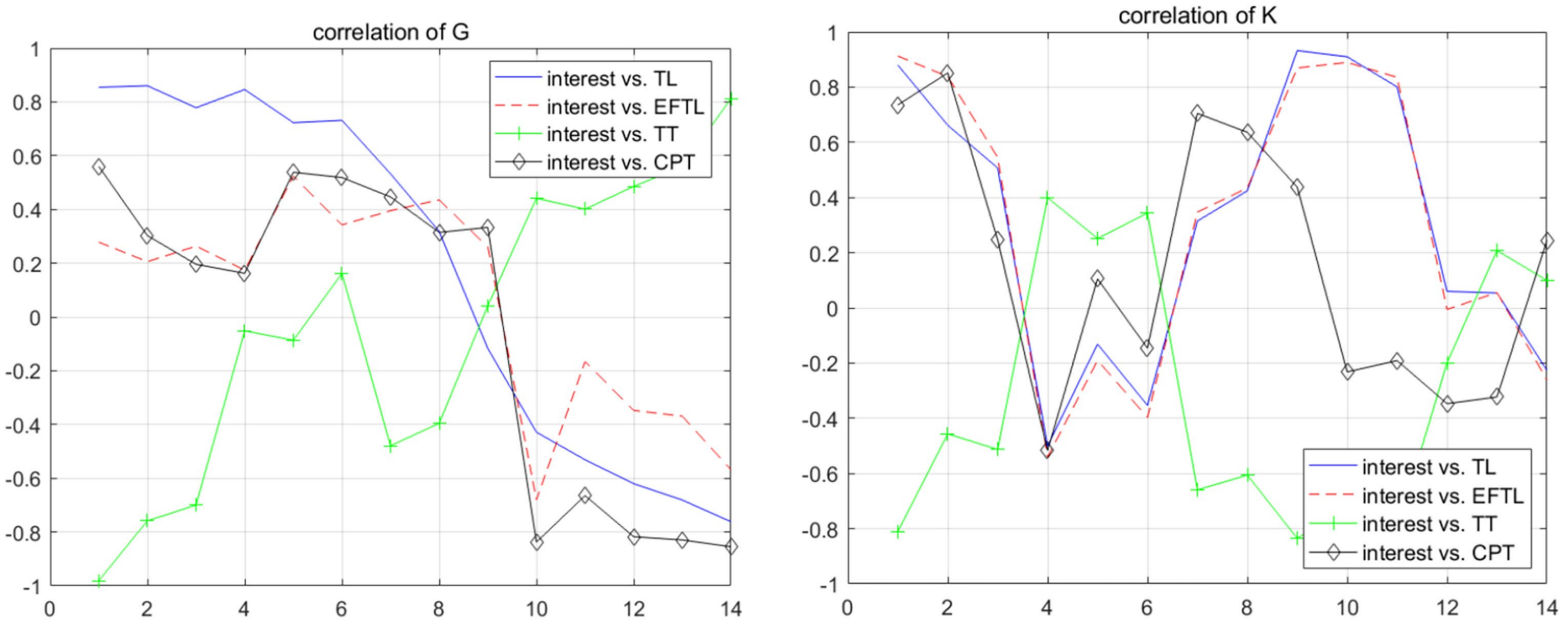
Correlation development curves of students G and K.

The effects of topic interest on the various components of the fluency system exhibit both synergistic and divergent characteristics. In this study, synergistic development is defined as a scenario where the trends of the two fluctuation curves and their positions on the vertical axis are roughly aligned. This synergy is primarily observed in the dynamic interaction between topic interest and the parameters of the lexical fluency subsystem. Among the 17 students, the dynamic change curves for the relationships between topic interest and TL, as well as topic interest and EFTL, showed a high degree of convergence for 8 students. As illustrated in Figure 2, the curves for Student K are nearly overlapping, reflecting the synergistic development of components within the system (Zheng and Feng, 2017; Li, 2023).

In addition to the synergistic development observed within the lexical fluency subsystem, there is also a notable convergent trend between the lexical and syntactic subsystems. For 3 students, the development curves for the relationship between a lexical fluency indicator (TL or EFTL) and topic interest closely align with the curves for the relationship between CPT and topic interest. For example, in Figure 2, the curves for Student G depicting the relationships between EFTL and topic interest, as well as CPT and topic interest, are highly overlapping. This demonstrates that lexical fluency and syntactic fluency, as subsystems of written language fluency, can mutually reinforce and develop together under the influence of the same factor—topic interest.

The synergistic development patterns observed in this study can be categorized into four main types. Among the students, 47.06% exhibited synergistic development only between the two lexical fluency parameters (TL and EFTL), while 5.88% showed synergistic development solely between lexical fluency parameters and the syntactic fluency parameter (CPT). Additionally, 11.76% demonstrated synergistic development both within the lexical fluency subsystem and between lexical fluency and CPT, whereas 35.29% did not exhibit any synergistic development. These findings highlight that synergistic development within the fluency system is a relatively common phenomenon, reflecting the interconnected and dynamic nature of its subsystems.

The differential effects of topic interest on the fluency system are primarily evident in two aspects: (1) the interaction between factors within the syntactic subsystem and (2) the interaction between the syntactic and lexical subsystems. For nearly all students, when topic interest promotes the development of TT, it tends to inhibit the growth of CPT or factors within the lexical subsystem. For example, in the case of Student K, when topic interest exhibits a negative correlation with TT in moving windows 1–3 and 7–9, it simultaneously shows a positive correlation with TL, EFTL, and CPT. This pattern highlights the imbalance in resource allocation within the fluency system and underscores the divergent developmental trajectories of its various components.

Under the influence of changes in topic interest, the fluency subsystems exhibited both synergistic and divergent patterns, both within and between subsystems. These findings align with the research of Li (2023, 2024a), which highlights the alternating dynamics of supportive and competitive relationships within the system. When resources are scarce and developmental demands increase, competitive relationships tend to dominate. However, when resource allocation within the system stabilizes, internal factors support one another and progress together.

According to the findings of this study, under the influence of topic interest, coordinated development is most prevalent within the lexical fluency subsystem, followed by between the lexical and syntactic subsystems, and least common within the syntactic subsystem itself. This result contrasts with the findings of Zheng and Feng (2017), who investigated the correlations within and between subsystems of written syntactic and lexical complexity among advanced Chinese learners of English. Their study revealed that factors within a single subsystem primarily exhibited mutual support, while significant competitive relationships were observed between subsystems. In contrast, this study demonstrates that significant supportive relationships are not necessarily confined within a single subsystem and that coordinated development can also occur between subsystems. The discrepancy between these two studies underscores the complex and dynamic nature of complex dynamic systems. Differences in the target language, learner proficiency levels, and the specific subsystems examined may contribute to these inconsistent findings. In addition, This finding suggests that teachers can help students balance lexical and syntactic attention during writing tasks, thereby promoting more coordinated development between fluency subsystems.

From a psycholinguistic perspective, the synergistic and divergent effects of topic interest on the fluency system essentially reflect two key characteristics of the L2 cognitive system: subsystem interaction and dynamic resource reorganization. The high convergence within lexical fluency (e.g., overlapping curves for TL and EFTL in Student K’s case) stems from their shared core cognitive foundation—lexical retrieval automaticity. When topic interest activates topic-related lexical networks (Snellings et al., 2004), both lexical unit length (TL) and error-free lexical unit length (EFTL) benefit simultaneously, as both indicators rely on low-cognitive-load lexical retrieval processes and thus can improve in tandem without additional resource allocation.

In contrast, the divergent correlation between lexical and syntactic fluency (e.g., topic interest boosting TL while inhibiting CPT) reflects a competitive resource allocation mechanism. According to de Bot et al.’s (2007) dynamic cognitive theory of L2 acquisition, L2 subsystems follow an interactive logic of resource trade-off: when topic interest directs resources toward automatic lexical tasks, the controlled cognitive resources needed for syntactic encoding (e.g., clause integration, grammatical rule application) are further compressed, limiting the development of syntactic complexity (CPT). This pattern—where synergy depends on shared cognitive foundations and divergence arises from resource competition—not only reveals the underlying cognitive pathway through which topic interest influences fluency subsystems but also confirms the psycholinguistic characteristic of nonlinear subsystem interaction in the L2 cognitive system.

### 4.4 Phased development

The moving windows are divided into three stages based on the semester in which the writing activities occurred: Stage 1 includes moving windows 1, 2, and 3, with all writing activities taking place in the first semester; Stage 2 comprises moving windows 4, 5, 6, and 7, where writing activities span both semesters; and Stage 3 includes moving windows 8, 9, 10, 11, 12, 13, and 14, with all writing activities occurring in the second semester. Table 4 summarizes the total number of students exhibiting staged development, the total number of change points, and the distribution of these change points across the three stages for the four correlation dimensions.

**Table 4:** Summary of change point distribution.

Correlation dimensions	Number of students	Number of change points	First stage	Second stage	Third stage
Interest & TL	8	8	1	1	6
Interest & EFTL	10	13	1	3	9
Interest & TT	13	15	0	7	8
Interest & CPT	11	14	0	6	8

The data in Table 4 reveal that most students exhibit staged development in their fluency. As the learning stages progress, the interaction patterns between topic interest and the fluency system undergo qualitative changes, primarily occurring in the second or third stages. This reflects a degree of stability and continuity in the interaction between topic interest and the fluency system. From the initial stages of interaction, although the correlation coefficients fluctuate within a narrow range, they maintain a stable and consistent interaction pattern throughout the first semester. It is only in the middle and later stages that significant qualitative changes emerge.

In addition, the transformation trends at the change points between topic interest and fluency parameters can be categorized into six types: (1) a significant increase in positive correlation, (2) a significant weakening of positive correlation, (3) a shift from positive to negative correlation, (4) a significant increase in negative correlation, (5) a significant weakening of negative correlation, and (6) a shift from negative to positive correlation. The distribution of students exhibiting these six transformation types is detailed in Table 5.

**Table 5:** Summary of change point trend distribution.

Change point trend	Interest & TL	Interest & EFTL	Interest & TT	Interest & CPT
Positive↑	37.5%	23.08%		7.14%
Positive↓		7.69%	6.67%	
Positive -negative	50%	38.46%	26.69%	35.72%
Negative↑		7.69%	26.69%	21.43%
Negative↓				7.14%
Negative–positive	12.5%	23.08%	40%	28.57%

As shown in Table 5, significant commonalities are observed within both the lexical and syntactic fluency subsystems. Within the lexical fluency subsystem, the more prominent trends in the correlation between TL and topic interest—such as a significant increase in positive correlation and a shift from positive to negative correlation—are also the most prominent trends in the correlation between EFTL and topic interest. Similarly, the trends that are absent or negligible in the TL and interest correlation, such as a decrease in positive correlation and an increase or decrease in negative correlation, are also either absent or minimally represented in the EFTL and interest correlation.

Parallel patterns are evident within the syntactic fluency subsystem. In the correlations between TT and topic interest, as well as between CPT and topic interest, the most prominent trends include a shift from positive to negative correlation, an increase in negative correlation, and a shift from negative to positive correlation.

Both commonalities and differences exist between the lexical and syntactic fluency subsystems. The commonalities are primarily reflected in the fact that, across all four correlations, the shifts from positive to negative correlation and from negative to positive correlation are prominent trends. The differences, however, lie in the proportion of students exhibiting the other trends. Specifically, the proportion of students showing an enhanced positive correlation is significantly higher in the lexical fluency subsystem than in the syntactic subsystem. Conversely, the proportion of students with an enhanced negative correlation is significantly higher in the syntactic subsystem than in the lexical subsystem. For example, in the correlations between TL and topic interest and between EFTL and topic interest, the trend of enhanced positive correlation accounts for 37.5 and 23.08%, respectively. In contrast, in the syntactic dimensions of TT and CPT, these proportions are only 0 and 7.14%. Similarly, the trend of enhanced negative correlation accounts for 26.69 and 21.43% in the correlations between TT and topic interest and between CPT and topic interest, but only 0 and 7.69% in the lexical fluency dimensions.

These findings demonstrate that, as subsystems of the fluency system, lexical fluency and syntactic fluency exhibit shared developmental patterns. However, the internal factors within the lexical fluency subsystem are more closely interrelated and exhibit greater commonality. According to Zheng and Feng (2017), shared developmental patterns can serve as a criterion for determining whether different parameters belong to the same (sub)system. Thus, this study validates the classification criteria that TL and EFTL belong to the lexical fluency subsystem, while TT and CPT belong to the syntactic fluency subsystem.

During the transitional phase of a dynamic complex system, it represents an optimal period for teachers to intervene in foreign language instruction (Li, 2023, 2024b). Furthermore, students’ positive learning beliefs, particularly their accurate understanding of the significance of written language fluency, play a crucial role in enhancing written language parameters (Li, 2023, 2024a, 2024b). Consequently, as the correlation between topic interest and fluency systems evolves in stages—typically toward the end of the first and second semesters—teachers are advised to conduct preliminary research. This preparation enables them to provide students with more structured topic-based essays that align with their interests. Simultaneously, increasing exposure to texts rich in advanced vocabulary and syntactic complexity, while emphasizing the importance of written language fluency, can significantly improve fluency-related metrics. Such efforts not only enhance written language proficiency but also strengthen the positive correlation between topic interest and the fluency system.

Psycholinguistically, the phased correlation changes between topic interest and fluency stem from coordinated evolution of learners’ cognitive abilities and resource strategies. In Stage 1 (first semester), limited vocabulary and low lexical automaticity restrict topic interest’s impact, with resources focused on basic vocabulary. By Stages 2–3, lexical automaticity frees resources, but growing syntactic complexity (e.g., clauses, tenses) increases controlled demands, creating conflicts—topic interest boosting lexical fluency may inhibit syntax (e.g., Student K, windows 7–9). This shows resource allocation in L2 writing adjusts with proficiency, and the automatic-controlled trade-off reconstructs with subsystem needs, providing a psycholinguistic basis for understanding L2 fluency’s phased cognitive development.

## 5 Conclusion

Guided by CDST, this study employs a longitudinal approach to investigate written language fluency among Spanish majors at a Chinese public university. Over a 1-year period, the research analyzed lexical and syntactic fluency parameters across 18 essays written by each participant, while also assessing their interest in the topics of these essays. The moving correlation coefficient between topic interest and fluency parameters was computed using the software Palabra XYZ.

The findings reveal several key patterns. First, the moving correlation coefficient exhibits continuous and complex fluctuations, with overall trends categorized into four distinct patterns: rising, falling, rising-then-falling, and falling-then-rising. Second, while the correlation between topic interest and fluency parameters often reaches statistical significance, these significant periods are relatively brief. Third, the impact of topic interest on fluency parameters demonstrates both synergistic and differential effects. Finally, most students exhibited stage-like development, with transition points predominantly occurring between the first and second semesters and during the second semester itself. Notably, significant differences were observed between the lexical and syntactic fluency subsystems in terms of overall trend distribution, types of significant correlations, collaborative development patterns, and transition point trends. These disparities highlight the uneven development of fluency subsystems and variations in resource allocation between them.

From the perspective of research content, prior studies (Wang et al., 2015; Li and Cai, 2022) have predominantly explored the factors influencing written language production through qualitative approaches, often without a focused analysis of specific factors, thereby lacking depth. In contrast, this study investigates the evolving impact of topic interest on written language fluency over time, employing quantitative methods to provide a more objective and visual representation of this influence. Furthermore, while most research on written language fluency grounded in CDST has focused on single fluency indicators, this study adopts a more comprehensive approach by examining four key indicators across the lexical and syntactic fluency subsystems. As a result, this study contributes to the field through its innovative research content and analytical methods, offering deeper insights into the nonlinear processes of written language production and their dynamic interactions with influencing factors.

Moreover, this study offers valuable insights for foreign language teaching practices. First, it highlights significant individual differences in learners’ topic interests and the interaction patterns between topic interest and written language fluency. Consequently, foreign language instruction should take these individual characteristics into full account, adopting tailored teaching strategies that cater to students’ unique aptitudes and preferences. Second, the findings reveal that during the first year of college, most students exhibit a significant positive correlation between topic interest and vocabulary fluency, alongside a significant negative correlation with syntactic fluency. This suggests that topic interest primarily drives the development of vocabulary fluency. Therefore, to foster the growth of the syntactic fluency subsystem, it is essential to enhance syntactic fluency input and reinforce students’ learning beliefs, such as raising their awareness of the importance of syntactic fluency. Finally, the correlation between topic interest and the written language fluency system often develops in stages, particularly toward the end of the first semester and throughout the second semester. During these critical periods, increasing the input of vocabulary and syntactic fluency, as well as strengthening related learning beliefs, can promote the development of the fluency system. This approach can help leverage topic interest to its fullest potential, facilitating a shift toward a positive correlation between topic interest and the fluency system.

This study focuses on essays written by Chinese Spanish majors during their first year of college as the primary research material. However, students’ Spanish writing skills undergo significant development throughout their four-year undergraduate program. How will written language fluency and its interaction patterns with topic interests evolve over this extended period? Will these patterns resemble those observed in the first year? These questions hold substantial academic value and will form the basis of our future research endeavors. Future studies could extend the current longitudinal design across multiple academic years to examine the stability and developmental trajectories of the observed interaction patterns. The current study has established a solid theoretical and methodological foundation for investigating written language fluency among senior students, paving the way for further exploration in this area.

## Data Availability

The raw data supporting the conclusions of this article will be made available by the authors, without undue reservation.

## References

[ref1] Abdel Latif M. M. M. (2009). Toward a new process-based indicator for measuring writing fluency: evidence from L2 writers' think-aloud protocols. Can. Mod. Lang. Rev. 65, 531–558. doi: 10.3138/cmlr.65.4.531

[ref2] Abdel Latif M. M. M. (2013). What do we mean by writing fluency and how can it be validly measured? Appl. linguist. 34, 99–105. doi: 10.1093/applin/ams073

[ref3] Al-Abri A. Ranjbaran Madiseh F. Morady Moghaddam M. (2024). Exploring learning-oriented assessment in enhancing students’ lexical fluency through MALL. Asia-Pac. Educ. Res. 34, 1–13. doi: 10.1007/s40299-024-00832-7

[ref4] Alisaari J. Heikkola L. M. (2016). Increasing fluency in L2 writing with singing. Stud. Second Lang. Learn. Teach. 6, 271–292. doi: 10.14746/ssllt.2016.6.2.5

[ref5] Baba K. Nitta R. (2014). Phase transitions in development of writing fluency from a complex dynamic systems perspective. Lang. Learn. 64, 1–35. doi: 10.1111/lang.12033

[ref6] Bertalanffy L. (1968). General system theory: Foundations, development, applications. New York: George Braziller Inc.

[ref7] Biria R. Jafari S. (2013). The impact of collaborative writing on the writing fluency of Iranian EFL learners. J. Lang. Teach. Res. 4, 164–175. doi: 10.4304/jltr.4.1.164-175

[ref8] Bruton D. L. 1986 Toward defining written fluency: connecting product and process (composing, schools)

[ref9] Chang C. Chang H. (2020). Guonei eryu xiezuo yanjiu huigu yu qianjing zhanwang [review and perspective of research on second language writing in China]. Waiyu Dianhua Jiaoxue 42, 61–67,10. doi: 10.20139/j.issn.1001-5795.2020.03.008

[ref10] Chenoweth N. A. Hayes J. R. (2001). Fluency in writing: generating text in L1 and L2. Written Commun. 18, 80–98. doi: 10.1177/0741088301018001004

[ref11] Dai Y. Wang T. (2012). Jiyu Dongtai xitong lilun de eryu xide Moshi yanjiu [a study on the second language acquisition model based on dynamic systems theory]. Shandong waiyu jiaoxue 21, 36–42. doi: 10.16482/j.sdwy37-1026.2012.05.018

[ref12] de Bot K. (2008). Introduction: second language development as a dynamic process. Mod. Lang. J. 92, 166–178. doi: 10.1111/j.1540-4781.2008.00712.x

[ref13] de Bot K. Lowie W. Verspoor M. (2005). Second language acquisition: an advanced resource book. London: Routledge.

[ref14] de Bot K. Lowie W. Verspoor M. (2007). A dynamic systems theory approach to second language acquisition. Bilingualism Lang. Cogn. 10, 7–21. doi: 10.1017/S1366728906002732

[ref15] Dörnyei Z. (2014). Researching complex dynamic systems: ‘Retrodictive qualitative modelling’in the language classroom. Lang. Teach. 47, 80–91. doi: 10.1017/S0261444811000516

[ref16] Fellner T. Apple M. (2006). Developing writing fluency and lexical complexity with blogs. Jalt Call J. 2, 15–26. doi: 10.29140/jaltcall.v2n1.19

[ref17] Feng L. Li K. X. Wang C. L. (2022). Dongtai xitong lilun shijiao xia sanyu xuexizhe jufa fuzadu fazhan bianyi tezheng gean yanjiu [a case study on the developmental variation characteristics of syntactic complexity among trilingual learners from the perspective of dynamic systems theory]. Xian waiguoyu daxue xuebao 30, 46–52. doi: 10.16362/j.cnki.cn61-1457/h.2022.04.015

[ref18] Foster P. (2020). Oral fluency in a second language: a research agenda for the next ten years. Lang. Teach. 53, 446–461. doi: 10.1017/S026144482000018X

[ref19] Hilton H. (2008). The link between vocabulary knowledge and spoken L2 fluency. Lang. Learn. J. 36, 153–166. doi: 10.1080/09571730802389983

[ref20] Hiver P. Al-Hoorie A. H. Evans R. (2022). Complex dynamic systems theory in language learning: a scoping review of 25 years of research. Stud. Second. Lang. Acquis. 44, 913–941. doi: 10.1017/S0272263121000553

[ref21] Housen A. Kuiken F. (2009). Complexity, accuracy, and fluency in second language acquisition. Appl. Linguist. 30, 461–473. doi: 10.1093/applin/amp048

[ref22] Johnson M. D. Mercado L. Acevedo A. (2012). The effect of planning sub-processes on L2 writing fluency, grammatical complexity, and lexical complexity. J. Second. Lang. Writ. 21, 264–282. doi: 10.1016/j.jslw.2012.05.011

[ref23] Kellogg R. T. (1996). “A model of working memory in writing” in The science of writing: Theories, methods, individual differences, and applications. eds. Levy C. M. Ransdell S. (Mahwah: Lawrence Erlbaum Associates, Inc).

[ref24] Kormos J. (2012). The role of individual differences in L2 writing. J. Second. Lang. Writ. 21, 390–403. doi: 10.1016/j.jslw.2012.09.003

[ref25] Kowal I. (2014). Fluency in second language writing: a developmental perspective. Stud. Linguist. Univ. Iagell. Cracov. 131, 229–246. doi: 10.4467/20834624SL.14.013.2321

[ref26] Kowal I. (2016). The dynamics of complexity, accuracy and fluency in second language development. Kraków: Jagiellonian University Press.

[ref27] Larsen-Freeman D. (1997). Chaos /complexity science and second language acquisition. Appl. Linguist. 91, 141–165. doi: 10.1093/applin/18.2.141

[ref28] Larsen-Freeman D. (2006). The emergence of complexity, fluency, and accuracy in the oral and written production of five Chinese learners of English. Appl. Linguist. 27, 590–619. doi: 10.1093/applin/aml029

[ref29] Larsen-Freeman D. Cameron L. (2008). Complex systems and applied linguistics. Oxford: Oxford University Press.

[ref30] Larsen-Freeman D. Schmid M. Lowie W. (2011). “Introduction: from structure to chaos. Twenty years of modeling bilingualism” in Modeling bilingualism: From structure to chaos. In honor of Kees de Bot. eds. Schmid M. Lowie W. (Amsterdam: John Benjamins Publishing Company).

[ref31] Larsen-Freeman D. y Cameron L. (2013). Complex systems and applied linguistics. Shanghai: Shanghai Foreign Language Education Press.

[ref32] Li J. (2023). Riqueza léxica en la producción escrita en español de alumnos chinos: un estudio basado en la teoría de los sistemas dinámicos complejos. Estudios de Lingüística 40, 293–310. doi: 10.14198/ELUA.24230

[ref33] Li J. (2024a). Desarrollo del lenguaje escrito en español como lengua extranjera en el contexto de estudio autónomo. Porta Linguarum 41, 119–136. doi: 10.30827/portalin.vi41.26608

[ref34] Li J. (2024b). Desarrollo dinámico de la motivación en una clase de español de alumnos universitarios chinos. Hip 107, 479–501. doi: 10.1353/hpn.2024.a948371

[ref35] Li J. Cai Y. (2022). El desarrollo dinámico de la expresión escrita de alumnos chinos que estudian la lengua española. Rasal Lingüística 19, 29–60. doi: 10.56683/rs222033

[ref36] Lorenz E. (1963). Deterministic non-periodic flow. J. Atmos. Sci. 20, 130–141. doi: 10.1175/1520-0469(1963)020<0130:DNF>2.0.CO;2

[ref37] Lowie W. (2017). “Lost in state apace? Methodological considerations in complex dynamic theory approaches to second language development research” in Complexity theory and language development: In Celebration of Diane Larsen-Freeman. eds. Ortega L. Han Z. (Amsterdam: John Benjamins).

[ref38] Lowie W. M. Verspoor M. H. (2019). Individual differences and the ergodicity problem. Lang. Learn. 69, 184–206. doi: 10.1111/lang.12324

[ref39] Ma R. Qin X. Q. (2013). Eryu xiezuo liulixing yanjiu qushi [research trends on second language writing fluency]. Xiandai Waiyu 36, 315–322.

[ref40] McAndrew D. A. (1990). Handwriting rate and syntactic fluency. J. Basic Writing 9, 31–39. doi: 10.37514/JBW-J.1990.9.1.04

[ref41] Mellon J. C. 1967 Transformational sentence-combining, a method for enhancing the development of syntactic fluency in English composition. Final report

[ref42] Nosratinia M. Razavi F. (2016). Writing complexity, accuracy, and fluency among EFL learners: inspecting their interaction with learners' degree of creativity. Theory Pract. Lang. Stud. 6, 1043–1052. doi: 10.17507/TPLS.0605.19

[ref43] Pan K. J. Yang L. R. Lu X. F. (2024). Zhongguo daxue yingyu xuexizhe cihui liulixing he fengfuxing de sunhao yanjiu [a study on the loss of vocabulary fluency and richness among Chinese college English learners]. Waiyu Jiaoxue Yu Yanjiu 56, 394–403. doi: 10.19923/j.cnki.fltr.2024.03.008

[ref44] Pham V. P. H. (2021). The effects of collaborative writing on students’ writing fluency: an efficient framework for collaborative writing. SAGE Open 11, 1–11. doi: 10.1177/2158244021998363

[ref45] Qin X. Q. Bi J. (2012). Eryu xiezuo liulixing zhibiao de xiaodu: yixiang jiyu wenben tezheng de yanjiu [validity of fluency indicators in second language writing: a study based on text features]. Waiyu Jiaoxue Yu Yanjiu. 44, 899–911+961.

[ref46] Rezazadeh M. Tavakoli M. Rasekh A. E. (2011). The role of task type in foreign language written production: focusing on fluency, complexity, and accuracy. Int. Educ. Stud. 4, 169–176. doi: 10.5539/ies.v4n2p169

[ref47] Rokoszewska K. J. (2022). The dynamics of monthly growth rates in the emergence of complexity, accuracy, and fluency in L2 English writing at secondary school–a learner corpus analysis. System 106:102775. doi: 10.1016/j.system.2022.102775

[ref48] Saadat M. Alavi S. Z. (2017). Inter-individual variability in CAF: a case study of two individuals and two pairs’ written productions. Int. J. Eng. Lang. Trans. Stud. 5, 61–74.

[ref49] Sadeghpour M. (2013). The impact of topic interest on second language reading comprehension. Int. J. Linguist. 5, 133–145. doi: 10.5296/ijl.v5i4.3981

[ref50] Schiefele U. Krapp A. (1996). Topic interest and free recall of expository text. Learn. Individ. Differ. 8, 141–160. doi: 10.1016/S1041-6080(96)90030-8

[ref51] Schmidt R. (1992). Psychological mechanisms underlying second language fluency. Stud. Second. Lang. Acquis. 14, 357–385. doi: 10.1017/S0272263100011189

[ref52] Shapiro D. A. (2011). Stuttering intervention: a collaborative journey to fluency freedom. Austin, Texas: Pro Ed.

[ref53] Smit D. W. (2004). The end of composition studies. Carbondale: Southern Illinois University Press.

[ref54] Snellings P. Van Gelderen A. De Glopper K. (2004). Validating a test of second language written lexical retrieval: a new measure of fluency in written language production. Lang. Test. 21, 174–201. doi: 10.1191/0265532204lt276oa

[ref55] Spoelman M. Verspoor M. (2010). Dynamic patterns in development of accuracy and complexity: a longitudinal case study in the acquisition of Finnish. Appl. Linguis. 31, 532–553. doi: 10.1093/applin/amq001

[ref56] Suzuki S. Kormos J. (2023). The multidimensionality of second language oral fluency: interfacing cognitive fluency and utterance fluency. Stud. Second Language Acquis. 45, 38–64. doi: 10.1017/S0272263121000899

[ref57] Tarone E. Downing B. Cohen A. Gillette S. Murie R. Dailey B. (1993). The writing of southeast Asian-American students in secondary school and university. J. Second. Lang. Writ. 2, 149–172. doi: 10.1016/1060-3743(93)90015-U

[ref58] Tian Y. Kim M. Crossley S. Wan Q. (2021). Cohesive devices as an indicator of L2 students' writing fluency. Read. Writ. 37, 419–441. doi: 10.1007/s11145-021-10229-3

[ref59] van Geert P. (1994). Dynamic systems of development: change between complexity and chaos. Birmingham: Harvester Wheatsheaf.

[ref60] Van Waes L. 2012 Using keystroke logging to better define writing fluency in L1 and L2. Paper presented at the 8th international symposium on EFL writing research and teaching in China. Shandong, China.

[ref61] Verspoor M. Lowie W. van Dijk M. (2008). Variability in second language development from a dynamic systems perspective. Mod. Lang. J. 92, 214–231. doi: 10.1111/j.1540-4781.2008.00715.x

[ref62] Wang H. H. Li B. B. Xu L. (2015). Zhongguo yingyu xuexizhe shumianyu shuiping fazhan gean Dongtai yanjiu [a case study on the development of written language proficiency of Chinese learners of English]. Waiyu Jiaoxue Yu Yanjiu 59, 67–80.

[ref63] Waninge F. Dörnyei Z. De Bot K. (2014). Motivational dynamics in language learning: change, stability, and context. Mod. Lang. J. 98, 704–723. doi: 10.1111/modl.12118, PMID: 41271573

[ref64] Wolfe-Quintero K. Inagaki S. Kim H.-Y. (1998). Second language development in writing: measures of fluency, accuracy, and complexity. Birmingham: University of Hawaii.

[ref65] Zhang S. M. (2009). The role of input, interaction and output in the development of oral fluency. Engl. Lang. Teach. 2, 91–100. doi: 10.5539/elt.v2n4p91

[ref66] Zheng Y.Y. (2019). Cong fuza Dongtai xitong lilun tan youxiao de waiyu jiaoxue. Dangdai waiyu yanjiu [on effective foreign language teaching from the perspective of complex dynamic system theory]. 19, 12–16+49.

[ref67] Zheng Y. Y. Feng Y. L. (2017). Xuexizhe jufa yu cihui fuzaxing fazhan de Dongtai xitong yanjiu [a dynamic systemic study on the development of learners' syntactic and lexical complexity]. Xiandai waiyu 40, 57–68+146.

[ref68] Zheng Y. Y. Li H. X. (2023). Fuza Dongtai xitong lilun shijiao xia eryu xiezuo fazhan de bianyixing yanjiu [a study on the variability of second language writing development from the perspective of complex dynamic systems theory]. Xiandai Waiyu 46, 650–663. doi: 10.20071/j.cnki.xdwy.20230620.006

[ref69] Zhou X. Y. 2024. Jiyu fuza Dongtai xitong lilun de liuxuesheng ketang yu zuoye kouyu nengli zhijian de bianyi yanjiu [a study on the variation between the oral ability of international students in class and on assignment based on complex dynamic systems theory]. Zhejiang keji daxue, MA thesis.

